# Clonidine is effective for the treatment of primary idiopathic hyperhidrosis and hot flushes: a case report

**DOI:** 10.1186/s13256-016-1174-2

**Published:** 2017-01-17

**Authors:** Ahmed Albadrani

**Affiliations:** Internal Medicine Department, College of Medicine, Prince Sattam bin Abdulaziz University, P.O. Box 173, Al-Kharj, 11942 Saudi Arabia

**Keywords:** Clonidine, Hyperhidrosis, Hot flushes, Case report

## Abstract

**Background:**

While primary hyperhidrosis can be seen in men, accompanying hot flushes is rarely seen in men. Primary hyperhidrosis is thought to be related to overactivity of the sympathetic nervous system while hot flushes are believed to be related to altered peripheral vascular reactivity and a narrowed thermoregulatory zone.

**Case presentation:**

I report the case of a 29-year-old man of Arab origin who presented to a dermatology clinic with a complaint of generalized sweating, with heavier involvement of his inguinal region, axilla, and lower back. His complaint was associated with a transient hot sensation and erythema over the affected areas. He did not respond to topical antiperspirants containing aluminum chloride, topical aluminum chloride, or to botulinum toxin A injected in both inguinal areas. He was then referred to an endocrinology clinic to rule out secondary causes of hyperhidrosis and hot flushes; a primary diagnosis was confirmed. He did not respond to oral glycopyrrolate and additionally was complaining of its anticholinergic side effects. The glycopyrrolate was then replaced with oral clonidine 0.15 mg twice a day. Clonidine was well tolerated without remarkable side effects and he quickly started to feel marked improvement which was maintained for 2 years.

**Conclusions:**

I report an atypical presentation of primary hyperhidrosis and hot flushes that was effectively controlled by clonidine without remarkable side effects. Further research on a large number of patients may be required before recommending clonidine in similar conditions.

## Background

Hyperhidrosis is a skin condition characterized by dysfunctional (excessive and unpredictable) sweating that often causes significant impairment of emotional and psychological well-being and may affect a person’s ability to carry out daily and occupational activities [1]. The prevalence of hyperhidrosis in the general population was estimated at approximately 3 %, with much higher prevalence among patients at a dermatology clinic [2, 3]. Primary (idiopathic) hyperhidrosis constitutes more than 90 % of the cases of hyperhidrosis and is diagnosed after excluding relevant underlying diseases or drug intake [4]. Common causes of secondary hyperhidrosis include endocrinal diseases, neurologic diseases, infection, alcoholism, and medications [4, 5]. The cause of primary hyperhidrosis is unknown, but is believed to be related to overactivity of the sympathetic nervous system in which acetylcholine is the neurotransmitter between a nerve ending and a sweat gland [1]. Another symptom that can be seen in relation with hyperhidrosis is hot flushes. They are characterized by a feeling of intense warmth, often accompanied by profuse sweating, anxiety, skin reddening, and palpitations, and sometimes followed by chills [6]. Hot flushes are believed to be caused by altered peripheral vascular reactivity and a narrowed thermoregulatory zone [7]. Hot flushes are well known in women: they affect approximately 75 % of menopausal women, with more than half of them seek medical advice [6]. However, the information about its prevalence in men and underlying etiology is very limited [8]. Hot flushes are mainly related to a sharp decline in the levels of testosterone as seen in patients with prostate cancer treated by androgen deprivation therapy [8]. However, rare medical conditions, some medications, and certain foods were associated with flushing of mainly the face and neck [9]. Here I report the diagnosis and the management of a rare case of hyperhidrosis and hot flushes in a patient with hypothyroidism.

## Case presentation

A 29-year-old man of Arab origin presented to a dermatology clinic with a complaint of increased sweating, with heavier involvement of his inguinal region, axilla, and lower back. The complaint was associated with a transient hot sensation and erythema over the affected area. Both complaints appeared for the first time 3 years ago and had progressed since then. His symptoms continued on a daily basis without diurnal variation and were aggravated by hot weather, caffeine, and anxiety. His sweating complaint was so severe (score 4 based on Hyperhidrosis Disease Severity Scale) that he had to change his clothes several times a day. This negatively affected his social interaction, quality of life, and the ability to work. He tried several over-the-counter antiperspirants with no significant improvement. He had no symptoms suggesting hyperthyroidism (including tremors, palpitation, and frequent bowel movements), tuberculosis/malignancy (fever, fatigue, loss of weight, and cough), or eczema/allergy (including skin changes, pruritus, and wheezing). He was not a tobacco smoker or alcohol user. He was single with no sexual history. He was socially and economically stable and living with family. He had no previous surgeries with the exception of an appendectomy when he was a teenager. He claimed that he was not taking any medications. His family history revealed no similar conditions and was negative for tuberculosis.

On examination, he was conscious and oriented with stable vital signs and without orthostatic changes. His chest was clear with no signs of infection or wheezing. Heart and blood vessels were normal with no signs of heart failure. His abdomen was soft and lax with no tenderness, organomegaly, or masses. His central nervous system was grossly intact. Joints were normal with no swelling or tenderness. There was no lymphadenopathy, jaundice, or pallor. Laboratory findings showed normal complete blood count, sedimentation rate, electrolytes, blood glucose, liver function, and renal function. A lipid profile showed normal low-density lipoprotein (LDL) and high-density lipoprotein (HDL) cholesterol and triglycerides but slightly high total cholesterol. A bone profile showed normal calcium, phosphate, alkaline phosphatase, and parathyroid hormone but low vitamin D. His uric acid just crossed the upper limit. Thyroid function tests showed normal levels of thyroxine (FT4, 13.8 pmol/L) and thyroid-stimulating hormone (TSH, 3.8 mU/L).

He was treated by topical antiperspirants containing aluminum chloride (Drysol) but without much improvement. To respond to his request of symptomatic treatment of the embarrassing inguinal hyperhidrosis, 50 U Botox (botulinum toxin A) was injected into each inguinal area after localization of sweating activity using a starch-iodine test. Injections were done using local anesthetic, approximately 1 to 2 cm apart, and were well tolerated with no complaint of pain or discomfort. He experienced some improvement in the inguinal hyperhidrosis for the first 3 months after the injections, but then the condition worsened.

He was then referred to an endocrinology clinic to rule out secondary causes of hyperhidrosis and hot flushes. He was reassessed and laboratory tests were repeated with no remarkable changes compared with the initial assessment. To evaluate endocrine causes of hyperhidrosis, a detailed hormonal profile was examined and showed normal thyroid function (FT4 and TSH), adrenal function (cortisol and dehydroepiandrosterone) and reproductive function (follicle-stimulating hormone, luteinizing hormone, testosterone, progesterone, estradiol, and prolactin), and insulin-like growth factor. In addition, to assess the presence of neuroendocrine tumors, a 24-hour urine collection was examined for 5-hydroxyindoleacetic acid and vanillylmandelic acid and his blood was examined for chromogranin. The results of the last three tests were high (95.1 umol/day, 104.4 umol/day, and 8.0 nmol/L, respectively), suggesting the presence of carcinoid or other neuroendocrine tumors. Therefore, a series of tests was done to confirm the presence and locate the site of such tumors. Computed tomography of his chest, abdomen, and pelvis was normal. A lower gastrointestinal endoscopy and bronchoscopy were normal. A whole body octreotide scan (somatostatin receptor scintigraphy) showed small focal increase of tracer uptake in his liver. However, single-photon emission computed tomography (SPECT) scan magnetic resonance imaging of his liver was unremarkable. In addition, repeated blood chromogranin A and 24-hour urine 5-hydroxyindoleacetic acid tests done 3 months later were normal (3.0 nmol/L and 26.2 umol/day, respectively).

After ruling out secondary causes, he was diagnosed as having primary generalized hyperhidrosis and hot flushes. The endocrinologist started my patient on oral glycopyrrolate (Robinul) 2 mg twice a day for 3 months. However, his hyperhidrosis and hot flushes did not improve and he was additionally complaining of anticholinergic side effects of glycopyrrolate such as dry mouth and constipation. The glycopyrrolate was then replaced with oral clonidine 0.15 mg twice a day. Clonidine was well tolerated without remarkable side effects. After he was started on clonidine he had regular blood pressure monitoring which showed no evidence of hypotension and he quickly started to feel marked improvement in both his hyperhidrosis and hot flushes (score 1 based on Hyperhidrosis Disease Severity Scale). According to the last patient contact, the improvement was still maintained on clonidine 2 years later without remarkable side effects.

## Discussion

I report a case of primary generalized hyperhidrosis and hot flushes that was effectively controlled by clonidine. The occurrence of hyperhidrosis and hot flushes in a young man is a rare presentation. Moreover, the atypical presentation of primary hyperhidrosis in the inguinal region is another rare location of primary hyperhidrosis which usually affects axillae, palms, soles, and craniofacial areas [4].

The primary hyperhidrosis diagnosis in my patient was confirmed after an exhaustive search of underlying causes such as diabetes, hyperthyroidism, pheochromocytoma, carcinoid syndrome, malignancy, tuberculosis, heart and lung diseases, alcohol intake, and relevant medications [4, 5]. Similar to this case, the onset of primary hyperhidrosis was frequently reported at the age of 25 years or younger and the presentation is usually bilateral, symmetric, and focal [4]. However, some characteristics frequently linked to cases of primary hyperhidrosis were lacking, such as positive family history and cessation of focal sweating during sleep [5]. Similarly, carcinoid or other neuroendocrine tumors, alcohol intake, and relevant medications were excluded as possible causes of hot flushes [9].

Clonidine in this report was more effective and tolerable than glycopyrrolate in relieving the symptoms of both hyperhidrosis and hot flushes. Clonidine is a centrally acting alpha adrenergic receptor agonist that reduces sympathetic stimulation, whereas glycopyrrolate inhibits synaptic acetylcholine and therefore interferes with neuroglandular signaling [10]. Although clonidine is not usually listed in the management of hyperhidrosis [5, 11], both drugs were recently shown to be effective as systemic treatment for primary hyperhidrosis [10]. However, oral medications for hyperhidrosis were only recommended after failure of topical aluminum chloride and botulinum toxin A for the fear of intolerability [11]. In addition, clonidine was shown to be an effective treatment for hot flushes in menopausal women [6]. The relief of both hyperhidrosis and hot flushes by clonidine may reflect the role of sympathetic stimulation in both condition and importance of central inhibition of sympathetic stimulation in concomitant presentation and refractory cases of hyperhidrosis.

## Conclusions

I here report a case with atypical presentation of idiopathic generalized hyperhidrosis and hot flushes that were effectively controlled by clonidine without remarkable side effects. Further research on a large number of patients may be required before recommending clonidine in similar conditions.

## Patient’s perspective

I was sweating constantly due to an unknown cause. I always had red skin, was hot and sweating all over my body especially my groin, armpits and lower back. I had to keep my air conditioning on at home 24/7 just so I wouldn't sweat. My doctor gave me clonidine 0.15 mg twice daily after non-effective topical management and almost immediately I noticed a difference. I have been on it for almost 2 years now and my sweating completely stopped and is under control. Thanks to clonidine the sweating has stopped and I can wear now whatever I want and be comfortable like a normal person without being completely drenched in sweat!!
